# The Clinical Profile of Newly Diagnosed Acute Myeloid Leukemia at a Tertiary Care Center in South India: A Cross-Sectional Study

**DOI:** 10.7759/cureus.61234

**Published:** 2024-05-28

**Authors:** Vandana G Hari, Naveenkumar Nallathambi, Vikram Y, Karthikeyan A, Shriganesh P Naidu

**Affiliations:** 1 Clinical Hematology, Madras Medical College, Chennai, IND; 2 Internal Medicine, Madras Medical College, Chennai, IND; 3 Hematology, Madras Medical College, Chennai, IND

**Keywords:** tumor-lysis syndrome, anemia, thrombocytopenia, chemotherapy agents, acute myeloid leukemia (aml)

## Abstract

Background and objective

Acute myeloid leukemia (AML) is a heterogeneous and aggressive blood malignancy prevalent among both children and adults, accounting for a significant proportion of acute leukemia cases worldwide. Our study aimed to shed light on the demographic and clinical profile and risk stratification of newly diagnosed AML cases at a tertiary care government hospital in South India.

Methods

We conducted a cross-sectional study involving 221 patients with AML in the Department of Clinical Hematology, Rajiv Gandhi Government General Hospital and Madras Medical College, Chennai, Tamil Nadu from January 2020 to December 2022. All data were collected from the hospital database of patients' medical records. A thorough analysis of clinical history, comorbidities, laboratories, risk stratification, and chemotherapy regimen was performed. The patients included in the study were newly diagnosed cases of AML over the age of 13 years, and we excluded all the relapsed cases.

Results

The highest proportion of patients were in the age group of 41-50 years (22.2%), and there was a significant male predominance (55.7%) in the cohort. Occupationwise, 31% of the study population were farmers, followed by housewives (16.3%). While no identifiable risk factors for AML were found in 191 cases (86.4%), 4.1% had undergone previous chemotherapy, and 3.6% had myelodysplastic syndrome (MDS). Hyperuricemia was noted in 50 cases (22.6%) while 8.6% had tumor lysis syndrome (TLS). About 53.8% of cases fell in the intermediate risk category of AML. Standard induction chemotherapy was administered in 87.3% of cases of AML.

Conclusions

Gaining awareness and knowledge about the regional demographic data and clinical presentation of AML will aid in the early detection, prompt referral, and initiation of treatment, thereby further improving patient outcomes in the era of targeted therapy and hematopoietic stem cell transplantation.

## Introduction

Leukemia is a hematological malignancy characterized by abnormal and excessive proliferation of malignant white blood cells, leading to their accumulation primarily in the bone marrow [1,2]. Acute myeloid leukemia (AML) is a rare, yet highly malignant neoplasm responsible for a large number of cancer-related deaths [3]. AML is a genetically very heterogeneous disorder characterized by the accumulation of somatically acquired genetic changes in hematopoietic progenitor cells that alter normal mechanisms of self-renewal, proliferation, and differentiation [4]. AML originates from myeloid lineage cells within the bone marrow due to chromosomal abnormalities, resulting in the accumulation of myeloblasts in the bone marrow and their infiltration into peripheral tissues [5].

The global incidence of AML is three to four cases per 100,000 population [6]. It is more frequently observed in males, with a male-to-female ratio of 2.5:1. AML accounts for 80-90% of acute leukemia in adults. The likelihood of its occurrence rises with advancing age. While there have been remarkable improvements in survival rates among younger patients, the prognosis remains very dismal for older individuals. Prior case-control studies have identified significant lifestyle and environmental factors linked to a heightened risk of AML, such as obesity, smoking, acetaminophen usage, and living in rural areas or farming communities. However, their influence on the clinical and genetic characteristics of AML remains unclear.

The clinical manifestations of AML arise from the infiltration of myeloblasts into both the bone marrow and extramedullary organs. Infiltration of tumor cells into the bone marrow causes symptoms of bone marrow failure, which presents as anemia, bleeding, infections, and fever [7]. Anemia presents with pallor and weakness, while fever is a common complaint among AML patients. Bleeding typically appears as petechiae, bruises, epistaxis, melaena, hematuria, and gum bleeding. Infiltration of tumor cells into the liver and spleen results in hepatosplenomegaly.

The French-American-British (FAB) group and the World Health Organization (WHO) have classified AML into various subtypes. The FAB classification, based on the morphology of myeloblasts and cytochemistry, has divided AML into eight subtypes, i.e., from M0 to M7 [8,9]. The current WHO classification of AML is based on genetic abnormalities, morphology, and immunophenotype. Treatment strategies are impacted by a range of factors, including patient characteristics such as age, comorbidities, BMI, and performance status, along with disease attributes, where the genetic profile of the disease stands out as the most critical prognostic factor. In India, AML is underdiagnosed, underreported, and causes a huge economic burden to the patients [10]. There is a relative paucity of studies regarding AML from India. In light of this, our study aims to shed light on the demographic and clinical profile, risk stratification, and hematological and biochemical parameters of newly diagnosed AML cases at a tertiary care government hospital in South India.

## Materials and methods

This was a cross-sectional study involving 221 patients diagnosed with AML at the Department of Clinical Hematology, Rajiv Gandhi Government General Hospital and Madras Medical College, Chennai, spanning the period from January 2020 to December 2022. After obtaining approval from the Institutional Ethical Committee, Madras Medical College (No-DHR-EC/NEW/INST/2021/1618), data were collected from the hospital database. The patient consent was not required since the study involved data from the hospital registry. The data collected included patient history, age, sex, occupation, comorbid conditions, clinical examination findings (hepatomegaly/splenomegaly/lymphadenopathy), hematological and biochemical data (WBC/hemoglobin/platelet/uric acid), AML risk category, risk stratification, and chemotherapy used. The inclusion criteria comprised newly diagnosed AML cases aged 13 years and above, ensuring a focus on primary occurrences. Relapsed cases and acute promyelocytic leukemia (APML) cases were excluded to maintain homogeneity within the study cohort. The collected data underwent comprehensive analysis, providing insights into the diverse aspects of AML.

The analysis utilized the European LeukemiaNet (ELN) 2022 risk assessment system to categorize patients into favorable, intermediate, or adverse risk groups based on their specific genetic mutations and chromosomal abnormalities [11]. This risk stratification helps predict patient outcomes and guide treatment decisions. To assess responses to treatment, bone marrow aspiration and biopsy were performed at specific time points, typically following count recovery or on day 28 post-chemotherapy, whichever came first.

Morphologic complete remission (CR) was defined according to established criteria, including less than 5% bone marrow blasts, undetectable peripheral blood blasts, and evidence of hematologic recovery [absolute neutrophil count (ANC) ≥1,000/μL and platelet count ≥100,000/μL). The refractory disease was defined as the failure to achieve CR following induction chemotherapy. By examining these parameters, the study aimed to enhance our understanding of AML epidemiology and thereby inform clinical decision-making.

## Results

The highest proportion of patients were in the age group of 41-50 years. Individuals aged below 20 years and those over 61 years comprised the smallest groups. The gender distribution was as follows: 44.3% females and 55.7% males. Regarding occupation, farmers accounted for 31.7%, followed by students and office workers (both 14.0%). The majority of cases had no identified risk factors (86.4%). Among those with identified risk factors, the most common ones were chemotherapy (4.1%) and myelodysplastic syndromes (MDS) (3.6%). Hyperuricemia was present in 22.6% of cases, while tumor lysis syndrome (TLS) was seen in 8.6%. Risk classification showed that the majority of cases fell into the intermediate-risk category (53.8%). Of note, 24.9% of cases were classified as high-risk. The most common treatment regimen was the "7+3 regimen" (87.3%). Azacitidine and low-dose cytarabine represented less common treatment options, used in 9.0% and 3.6% of cases respectively. After induction therapy, 33.0% of cases achieved complete remission (CR), 32.6% of cases were refractory to treatment, and 34.4% of cases expired (Table 1).

**Table 1:** Demographic characteristics and clinical risk factors AML: acute myeloid leukemia; BMF: bone marrow fibrosis; MDS: myelodysplastic syndrome; MPN: myeloproliferative neoplasm; TLS: tumor lysis syndrome

Variables	Categories	No. of cases	Percentage
Age group, years	<20	26	11.80%
	21-30	34	15.40%
	31-40	47	21.30%
	41-50	49	22.20%
	51-60	40	18.10%
	>61	25	11.30%
Sex	Male	123	55.70%
	Female	98	44.30%
Occupation	Housewife	36	16.30%
	Farmer	70	31.70%
	Student	31	14.00%
	Office work	31	14.00%
	Daily wage laborer	20	9.00%
	Driver	12	5.40%
	Small business	15	6.80%
	Teacher	4	1.80%
	Nurse	2	0.90%
Risk factors	None	191	86.40%
	BMF	0	0.00%
	Down syndrome	3	1.40%
	Previous chemotherapy	9	4.10%
	MDS	8	3.60%
	MPN	5	2.30%
	Radiation	1	0.50%
	Native medications	4	1.80%
Hyperuricemia	Yes	50	22.60%
	No	171	77.40%
TLS	Yes	19	8.60%
	No	202	91.40%
AML risk category	Favorable	19	8.60%
	Intermediate	119	53.80%
	High	55	24.90%
	Unclassified	28	12.70%
Treatment	7+3 regimen	193	87.30%
	Azacitidine	20	9.00%
	Low-dose cytarabine	8	3.60%
	Hydroxy urea	0	0.00%
Outcome after induction therapy	Complete remission	73	33.00%
	Refractory	72	32.60%
	Expired	76	34.40%

The majority (59.7%) had no recorded comorbidities. Among those with reported conditions, diabetes was the most prevalent (27.6%), followed by hypertension (9.0%), coronary artery disease (6.3%), chronic kidney disease (4.1%), pregnancy (3.2%), and rheumatic heart disease (0.5%). These findings highlight the prevalence of diabetes as a significant health concern, along with hypertension and cardiovascular conditions (Table 2).

**Table 2:** Comorbidities in the study population

Comorbidities	No. of cases	Percentage
None	132	59.7%
Diabetes	61	27.6%
Hypertension	20	9.0%
Coronary artery disease	14	6.3%
Chronic kidney disease	9	4.1%
Pregnancy	7	3.2%
Rheumatic heart disease	1	0.5%

Fever was the most common presentation, affecting 48.4% (107) of cases. Bleeding was observed in 20.4% (n=45) of cases, while fatigue was reported in 32.6% (n=72). Body pain was noted in 6.8% (n=15) of cases, followed by features of hyperviscosity (2.3%). Pulmonary embolism, neurological symptoms, and abdominal pain were less frequent, each representing less than 1% of cases (Table 3).

**Table 3:** Clinical presentation of the study population

Clinical presentation	No. of cases	Percentage
Fever	107	48.4%
Bleeding	45	20.4%
Fatigue	72	32.6%
Body pain	15	6.8%
Hyperviscosity	5	2.3%
Pulmonary embolism	0	0.0%
Neurological symptoms	2	0.9%
Abdominal pain	2	0.9%

On clinical examination, 47.1% (n=104) of cases showed no abnormalities. However, lymphadenopathy was observed in 12.2% (n=27) of cases, while hepatomegaly was present in 22.2% (n=49). Splenomegaly was noted in 37.1% of cases, indicating a significant proportion of patients with enlarged spleens. Neurological abnormalities observed were as follows: weakness of limbs due due cerebral bleed, meningitis, and cranial nerve palsy, seen in 1.8% (n=4) of cases; bone tenderness was less common, affecting only 0.9% of cases (Table 4).

**Table 4:** Clinical examination findings in the study population

Clinical findings	No. of cases	Percentage
None	104	47.1%
Lymphadenopathy	27	12.2%
Hepatomegaly	49	22.2%
Splenomegaly	82	37.1%
Neurological findings	4	1.8%
Bone tenderness	2	0.9%

**Figure 1:**
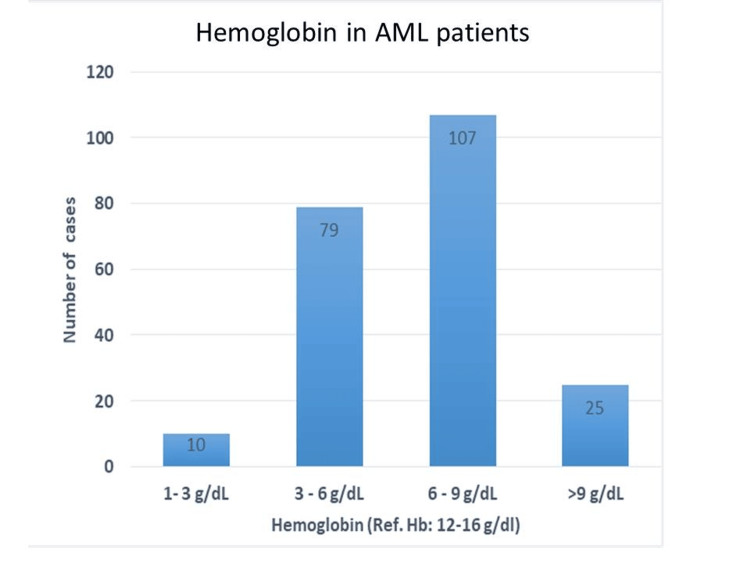
Hemoglobin levels in AML patients Hemoglobin reference range: 12-14 g/dL AML: acute myeloid leukemia

Hemoglobin exam showed that 4.52% had severe anemia (1-3 g/dl), 35.74% had moderate anemia (3-6 g/dl), and 48.41% had mild to moderate anemia (6-9 g/dl). Only 11.31% of cases had hemoglobin levels exceeding 9 g/dl. These findings emphasize a prevalent trend of anemia within the population, with a substantial portion exhibiting mild to moderate deficiency (Figure 1).

**Figure 2:**
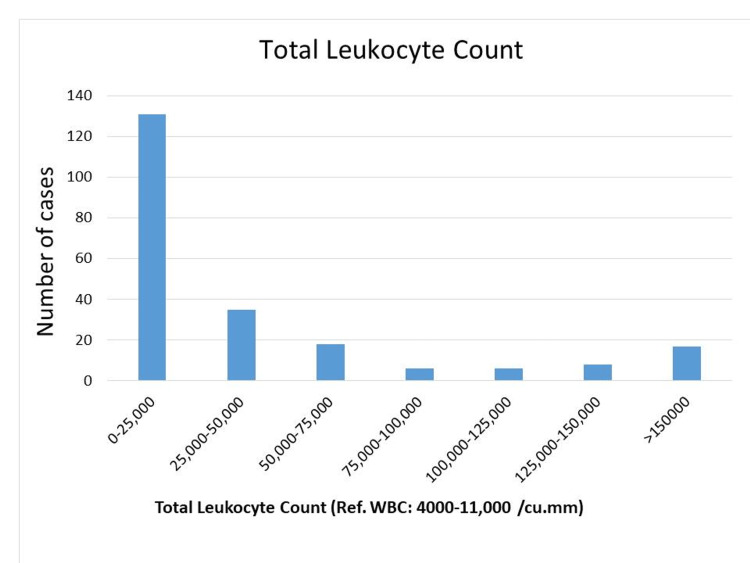
WBC count among the AML population WBC reference range: 4,000-11,000 per microlitre AML: acute myeloid leukemia; WBC: white blood cells

Total leukocyte count analysis revealed that 59.27% of cases had counts ranging from 0 to 25,000/cumm. Additionally, 15.83% had counts between 25,000 and 50,000/cumm, while 7.69% had counts exceeding 150,000/cumm. Counts between 50,000 and 150,000/cumm were less common and the incidence ranged from 2.71% to 8.14%. These findings indicate varying levels of leukocytosis, highlighting potential differences in immune response and underlying health conditions among individuals in the cohort (Figure 2).

**Figure 3:**
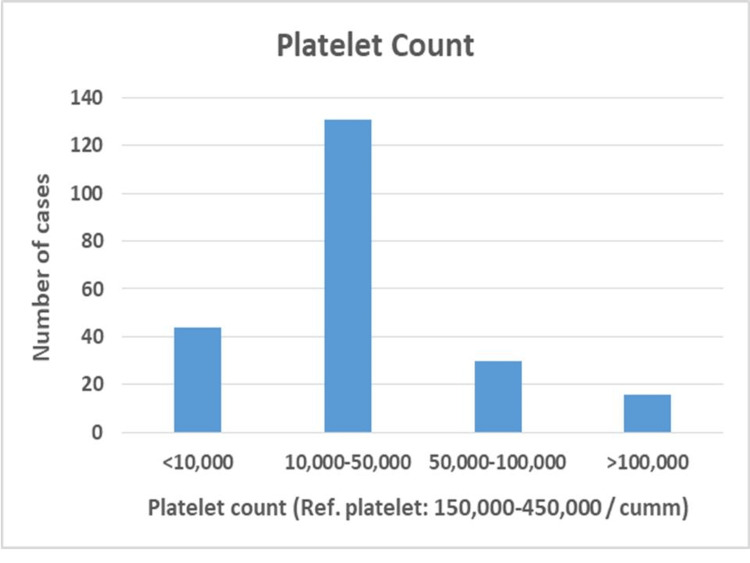
Platelet count in the study population Platelet reference range: 150,000-450,000 per microliter

Platelet counts varied in the studied population, with 35.82% exhibiting counts of 150,000-300,000/cumm, indicative of normal platelet levels. However, 27.92% showed thrombocytopenia (<150,000/cumm), while 36.25% had thrombocytosis (>300,000/cumm). These findings suggest that a notable proportion had abnormal platelet counts, highlighting potential hematological concerns and emphasizing the importance of further evaluation and management to address platelet-related conditions in this population (Figure 3).

## Discussion

AML is a belligerent hematological malignancy. It is characterized by an increase in the number of naive myeloid cells in the blood and bone marrow. These cells replace the normal cells and thereby harm the immune system. Historically, AML has been recognized as a predominantly male disease. Our study reinforces this observation, demonstrating a higher prevalence in males compared to females. These findings align with previous research by Shuchismita et al. and Acharya et al., potentially explained by the protective effects of hormonal variations in females as suggested by other studies [12-14].

Traditionally, AML has been associated with a higher incidence in the elderly population. However, our study observed a predilection for the 41-50 year age group. These results align with those of Acharya et al., and this finding warrants further investigation into the potential influence of environmental stressors, socioeconomic factors, and nutritional deficiencies. Our study found that a majority of patients hailed from an agricultural background, likely reflecting the predominant occupation in the local area. This observation coincides with the next finding that most patients lacked identifiable risk factors. While the use of chemical pesticides and fertilizers could be a potential risk factor for AML, further research is needed to establish a link, as suggested by the absence of definitive statistical evidence or documented associations [15].

The majority of our patients did not have any comorbidity. When present, the most frequent comorbidities were diabetes, followed by hypertension and coronary heart disease. These findings parallel those reported by Østgård et al. and Puumala et al. [16,17]. Comorbidities are known to adversely affect patient prognosis and decrease survival rates. The lower comorbidity burden in our study population might be due to their younger age compared to the typical age of comorbidity onset and potentially limited local awareness regarding these conditions. Similar to prior studies by Shuchismita et al. and Shaan et al., fever was the most frequent presenting complaint in our patients, prompting them to seek medical attention. In our study, clinical examinations yielded normal results in most cases. However, hepatomegaly and splenomegaly were the most common findings when abnormalities were present. These observations are consistent with those reported by Preethi et al., Chang et al., Asif et al., and Khan et al. [18]. The presence of these two conditions could be attributed to the infiltration of tumor cells in the organ in question. Pathologically diffuse infiltration of leukemic cells can be seen in the liver.

Analysis of blood cell counts revealed anemia to be the most frequent abnormality, with the majority of cases presenting with moderate anemia. Conversely, platelet levels were elevated in most patients, indicative of thrombocytosis. White blood cell counts typically fell within the range of 0-25,000/μL. These findings are consistent with those reported by Khan et al., Preethi et al., Asif et al., and Naeem et al. The underlying cause of these abnormalities lies in the mutations affecting hematopoietic genes characteristic of AML. These mutations lead to the clonal expansion of precursor cells, primarily undifferentiated myeloid precursors (blasts) detectable in the peripheral blood and bone marrow. This clonal expansion disrupts normal erythropoiesis, leading to ineffective red blood cell production and, ultimately, bone marrow failure. Consequently, the number of immature cells increases at the expense of mature, functional blood cells, representing the core pathogenesis of AML.

In our study, a significantly higher proportion (87.3%) of patients received standard induction therapy with cytarabine (100 mg/m^2^) and daunorubicin (60 mg/m^2^) compared to a similar study by Philip et al. (29%). This disparity highlights the critical role of accessible healthcare in ensuring optimal treatment for AML patients. High costs and lack of universal healthcare significantly limit access to these therapies [10]. The key cost drivers include hospitalization with associated medical expenses, stem cell transplantation for eligible patients, and the dynamic landscape of medication costs [19]. Our study observed a higher induction mortality rate (34.4%) compared to Radhakrishnan et al.'s study (13.78%) [20]. A risk classification proposed by Sasaki et al. has identified several factors associated with an increased risk of early mortality (within four weeks) in AML patients. These factors include older age, poorer performance status, elevated bilirubin levels, high creatinine levels, high uric acid levels, certain chromosomal abnormalities [excluding core-binding factor (CBF) and -Y translocations], and the presence of pneumonia [21].

Limitations

This study has a few limitations, primarily its single-center, cross-sectional observational design, and hence the findings of the study may not be generalizable to other populations or settings, particularly if the sample is not representative. More studies among people of varied ethnic backgrounds can help gain a better understanding of the disease.

## Conclusions

Our study found that AML patients in this region are typically male, diagnosed in their 40s, and often lack clear risk factors. Understanding these patient characteristics is crucial for early diagnosis and treatment, which is especially important with newer therapies that can significantly improve outcomes. This enhanced knowledge may enable healthcare providers to be more proactive in managing AML, potentially leading to better patient survival and quality of life. However, there is a significant gap in access to standard AML treatment in India, highlighting the need for broader efforts to improve healthcare access.
